# The 4AT scale for rapid detection of delirium in emergency department triage

**DOI:** 10.3389/fmed.2024.1345983

**Published:** 2024-05-14

**Authors:** Angela Soler-Sanchis, Francisco Miguel Martínez-Arnau, José Sánchez-Frutos, Pilar Pérez-Ros

**Affiliations:** ^1^Department of Nursing, Universitat de València, Valencia, Spain; ^2^Hospital Francesc de Borja, Generalitat Valenciana, Gandia, Valencia, Spain; ^3^Department of Physiotherapy, Universitat de València, Valencia, Spain

**Keywords:** delirium, aged, emergency service hospital, triage, data accuracy

## Abstract

**Aims:**

To assess the diagnostic accuracy and time impact of the 4AT scale in emergency department triage.

**Methods:**

A Prospective diagnostic accuracy study was carried out. People aged ≥65 years presenting to the emergency department from 1 November 2021 to 30 June 2022 were included. Nurses opportunistically screened eligible patients using the 4AT scale during triage according to the Manchester Triage System Francesc de Borja Hospital emergency department, Gandía (Spain). Accuracy was compared with medical diagnosis of delirium. Time (seconds) spent in triage with and without screening was assessed.

**Results:**

The study included 370 patients (55.1% men, mean age 81.8 years), of whom 58.4% (*n* = 216) were screened. A final diagnosis of delirium was made in 41.4% of those screened. The most frequently used presentational flow charts and discriminators were ‘behaving strangely’ (15%) and ‘rapid onset’ (33.3%). The highest accuracy was obtained for a score of 3 points or more (sensitivity 85.1%; specificity 66.9%; positive predictive value 52.8%; negative predictive value 71.7%). No significant differences were found in the time spent in triage according to the performance of screening.

**Conclusion:**

A score of 3 points or more on the 4AT scale enables rapid detection of delirium in emergency department triage, without consuming more time than conventional triage.

## 1 Introduction

Delirium is an acute neurobehavioral syndrome, characterized by acute and fluctuating disturbances of consciousness and attention in addition to possible disorientation, hallucinations, restlessness, confusion, and inappropriate behavior in hyperactive subtype or lethargy or increased sleepiness in hypoactive subtype (1). Research and clinical practice demonstrated that the development of delirium is multifactorial and involves a complex interrelationship between patient-, healthcare-and pharmacotherapy-related factors. The multifactorial nature is due to the concurrence of predisposing factors and precipitating factors. Age, cognitive deficit, drugs, sensory deficits, comorbidity and dehydration are some predisposing factors and urinary and respiratory infections and the administration or deprivation of psychotropic drugs and the administration of anticholinergic drugs as precipitating factors, as well as harsh hospital techniques (2). The syndrome has significant consequences for both the patient and the healthcare system, including higher rates of functional dependency and longer hospital stays, as well as increased risk of falling, institutionalization, morbidity, and mortality (3). Indeed, people with delirium carry almost three times the risk of death after hospital admission and at 6 months follow-up than those without (4–6).

Among older adults presenting to the emergency department (ED), an estimated 7 to 20% have delirium (1, 7, 8) with prevalence rising to 89% in people with pre-existing cognitive dysfunction or dementia (9). However the fluctuating nature of delirium results in under-diagnosis and under-treatment, with up to 83% cases being missed (3, 10).

There is a need to prioritize urgent care for all patients attending to ED. Triage is a method used to assess the severity of the patient’s condition and determine the level of priority for ED care. The nurse team is usually the responsible for this assessment. Every individual arriving at the ED requires an initial assessment, triage, which is conducted by the nursing team to determine and prioritize their care needs. The Manchester Triage System (MTS) is a systematized protocol to determine the patient’s severity as well as associated risks and needs, according to the flow chart, thus optimizing waiting time and resource use according to care needs (11). This process aims to provide a rapid and dynamic assessment (2, 12). Accurate and early detection of delirium may provide opportunities for identifying high-risk patients, potentially preventing or minimizing cases of delirium in the ED (9, 10, 13).

There are short, validated cognitive screening tools that could enable early identification of vulnerable older people, triggering appropriate care pathways and urgent assessment of people with possible delirium (4, 14). The most tools used are the 4 “A”s Test (4AT), Confusion Assessment Method (CAM), Confusion Assessment Method for the Intensive Care Unit (CAM-ICU), Brief Confusion Assessment Method (bCAM), 3-Minute Diagnostic Confusion Assessment Method (3D-CAM) and, Spatial Span Forwards (SSF), Clock Drawing Test (CDT) and Delirium Triage Screen (DTS). The common characteristic is that they are quick screening scales, generally requiring less than 3 min, suitable to be performed in the ED (15). Early detection and intervention in people with delirium is a strong indicator of the quality of hospital care for vulnerable patients (16, 17). Thus, this study aims to assess the accuracy of the 4AT scale, as administered by ED nurses in triage, and to compare time spent in triage between participants screened with the 4AT and those not screened.

## 2 Methods

### 2.1 Study design, setting and participants

This prospective diagnostic accuracy study included people aged 65 years or older who went to the ED between 1 November 2021 and 30 June 2022. Based on the findings of previous research the older persons with the highest risk of presenting delirium in ED were those with predisposing and precipitating factors such as dementia, previous stroke and infections or sedative drugs, respectively, in the triage assessment (18). In addition to patients who, after triage assessment following the MTS method by the nursing professional, are classified by flow charts ‘unwell adult’ or ‘abnormal behavior’, and/or discriminators ‘sudden onset’ or ‘a new neurological deficit less than 24 h old’ (11).

So, eligible patients presented predisposing and precipitating risk factors for delirium during triage by nursing professionals, (18) or were evaluated using the ‘unwell adult’ or ‘behaving strangely’ MTS flow charts with ‘rapid onset’ and ‘new neurological deficit less than 24 h old’ discriminators. The cognitive status of the patient with delirium is almost always compromised, so in such cases, the informed consent was signed by the family members of the participants. Participants who did not have family members to complete the informed consent form or decided not to take part in the study were excluded, as were people with delirium tremens or drug or substance intoxication.

All patients were seen in the ED at the Hospital Francesc de Borja de Gandía, Spain. This is a secondary, 256-bed, academic hospital with a catchment population of 188,000 and an average annual volume of 60,000 admitted ED encounters. The ED service is organized into eight care areas. In addition to the triage and admissions areas, the ED service has six care areas: consultations, resuscitation, observation, pediatrics, traumatology, and treatment room. Triage is performed by the nursing staff 24 h a day, 7 days a week. The ED is staffed by nine nurses during the morning and afternoon shifts, and seven nurses on the night shift. At least one nurse on each shift is responsible for triaging patients who come to the ED after being registered for emergency admission.

### 2.2 Sample size

A total of 8,426 people over 65 years of age attended the emergency department between November 1, 2021 and June 2, 2022. For a confidence level of 95% and a margin of error of 5%, a sample size of 368 participants was calculated.

### 2.3 Procedures

In 2011, the Edinburgh Delirium Research Group (Scotland, UK) developed the 4AT delirium screening scale, which consists of four items: an assessment of the level of alertness, an orientation test, an attention test, and finally an item determining acute change or fluctuating course (19). The instrument has since been translated to different languages and validated in multiple clinical settings, including the ED (20). The 4AT was used at first patient contact to rule out suspected delirium. It is an optimal tool for the ED because the estimated time for evaluation is <2 min. The Sensitivity is 89.7% and the specificity is 84.1% (21).

Following recruitment of the study cohort, participating nursing professionals opportunistically performed delirium screenings using the 4AT scale in the triage area, during all 7 days of the week and all three work shifts. Administration of the screening tool was contingent on having a sufficient number of professionals per shift, a manageable care load, and an acceptable time delay to receive health care.

**Figure 1:**
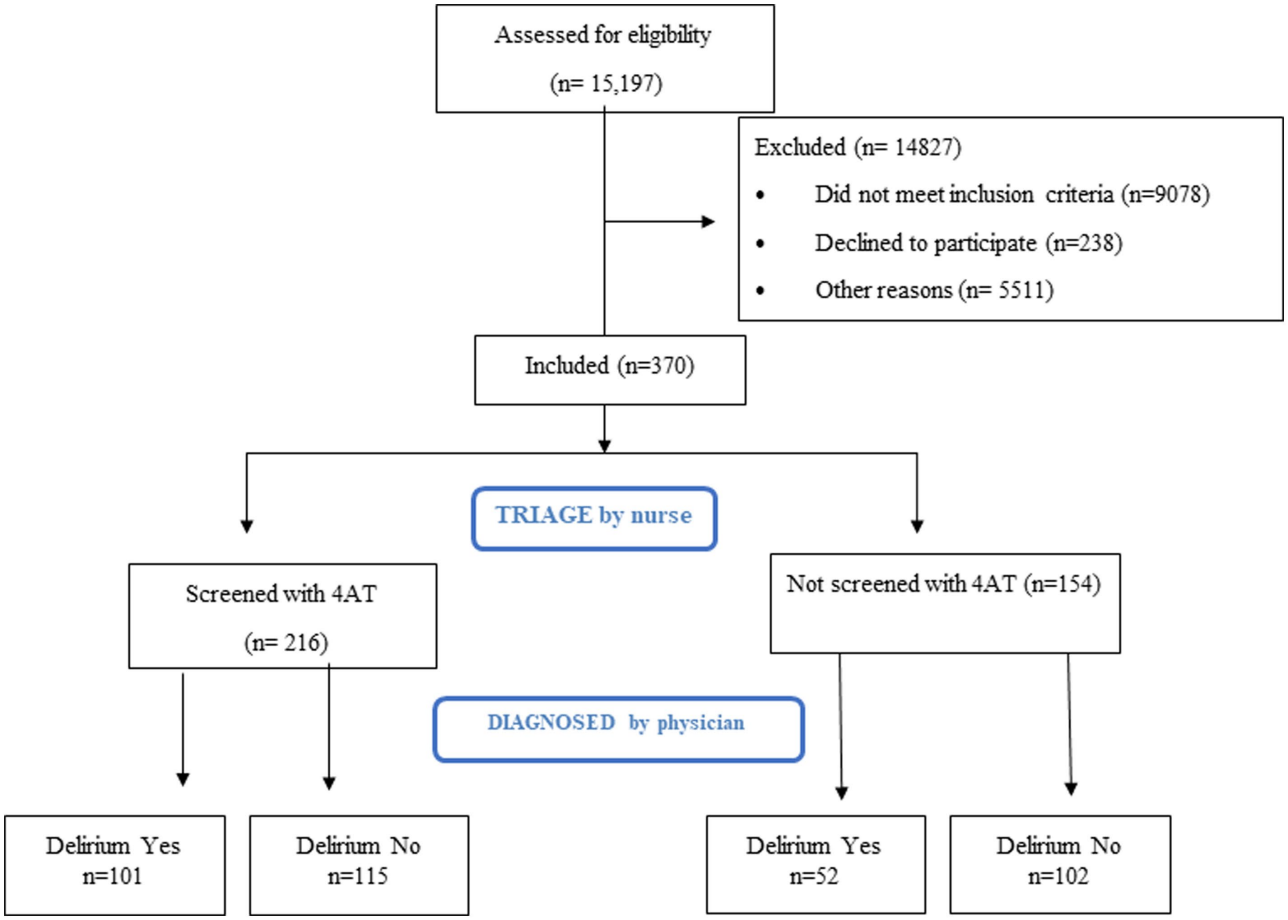
Study flow chart.

The results of the 4AT screening were added to each patient’s medical record as supplementary information for physicians. The diagnostic process was based on the DSM-V criteria, which include alteration of attention and consciousness, development over a short period of time, and additional changes in cognition and attention that are not attributable to a preexisting or developing neurocognitive disorder, or to a state of severe consciousness impairment (coma). Additionally, the 4AT score and the results of complementary tests were used to establish the patient’s final diagnosis, determining whether it was delirium or another condition. Following care, follow-up and diagnosis, the group screened with the 4AT scale were classified according to whether they received a medical diagnosis of delirium following DSM-V criteria (Screened with 4AT and Delirium Yes): disturbance in attention and awareness; develops over a short period of time; and additional disturbance in cognition, attention and cognition are not from a pre-existing or evolving neurocognitive disorder or from severely reduced arousal (coma) and those who were diagnosed with other pathology (Screened with 4AT and Delirium No). We also identified eligible patients who were not screened with the 4AT scale but were diagnosed with delirium (Not screened with 4AT and Delirium Yes), along with patients who fit selection criteria but did not have delirium, collecting data from their medical records following the ED episode (Not screened with 4AT and Delirium No) (Figure 1).

### 2.4 Data collection

In addition to collecting the results of the 4AT scale and the medical diagnosis using the ICD-10 code, we recorded sociodemographic data (age, sex) and comorbidities related to delirium (dementia, incontinence, history of stroke, and fall in the previous 30 days). Additionally, MTS variables were the presentational flow chart, discriminator, and priority, as well as the time spent by nurses in triage and the waiting time to be seen by the physician. Finally, the length of hospital stay in case of admission was collected.

### 2.5 Ethical considerations

The Hospital Francesc de Borja ethics committee approved the study. Patient confidentiality was preserved in line with Spanish legislation on the protection of personal data following Organic Law 3/2018, of 5 December, on Protection of Personal Data and Guarantee of Digital Rights. The study was carried out in accordance with the principles of the Declaration of Helsinki. All participants signed informed consent.

### 2.6 Statistical analysis

All data entered into the database were verified by an independent second person. Descriptive statistics were expressed as mean and standard deviation (SD) for normally distributed continuous variables and relative frequencies for categorical (qualitative) variables.

The risk of delirium according to comorbidities was quantified using the crude odds ratio (OR). The accuracy of the 4AT scale for diagnosing delirium was assessed according to the scale validation cutoff (≥4 points), using the medical diagnosis based on DSM-V criteria as a gold standard. In addition, the receiver operating characteristics (ROC) curve was used to determine the cutoff value and sensitivity, specificity, positive predictive value (PPV) and negative predictive value (NPV) of 4AT scores and delirium.

Data were entered in MS Excel spreadsheets, then imported for analysis to SPSS (version 28.0, IBM Corp. Released 2015. IBM SPSS Statistics for Windows, Version 28.0. Armonk, NY: IBM Corp).

## 3 Results

During the recruitment period, 53,110 patients were seen in the ED of the study center: 28.6% (*n* = 15,197) were aged 65 years or over, and 216 of these were screened with the 4AT scale by the triage nurses. Another group of 154 patients were included in the study because they met selection criteria but were not screened using the 4AT scale to analyse the accuracy of de 4AT.

Thus, a total of 370 participants were assessed: 41.4% (*n* = 153) were coded with a diagnosis of delirium according to the ICD-10, while the remaining 58.6% (*n* = 217) finally received a different diagnosis.

The sample was predominantly male, and participants’ mean age was 81.82 years, with significant differences between those diagnosed with delirium versus those who were not (84.04 years versus 80.25 years; mean difference [MD] 3.79, 95 confidence interval [CI] 2.14–5.43, *p* < 0.001). On the other hand, there were no significant differences between groups according to sex or priority (Table 1).

**Table 1:** Sociodemographic profile and Manchester triage variables.

	Delirium (*N* = 153)	No Delirium (*N* = 217)	Total (*N* = 370)	
	*n* (%)*	*n* (%)*	*n* (%)*	*p* value^†^
Age, years, mean (SD)	84.04 (7.68)	80.25 (8.11)	81.82 (8.14)	<0.001
Sex				
Male	77 (50.3)	127 (58.5)	204 (55.1)	0.12
Female	76 (49.7)	90 (41.5)	166 (44.9)	
Priority				
Level 2 (orange)	10 (6.5)	16 (7.4)	26 (7.0)	0.74
Level 3 (yellow)	95 (62.1)	126 (58.1)	221 (59.7)	
Level 4 (green)	48 (31.4)	74 (34.1)	122 (33.0)	
Level 5 (blue)	0 (0)	1 (0.5)	1 (0.3)	
MTC flow chart				
Unwell adult	84 (54.9)	125 (57.6)	209 (56.5)	0.002
Behaving strangely	23 (15.0)	11 (5.1)	34 (9.2)	
Others	46 (30.1)	81 (37.3)	127 (34.3)	
MTC Discriminators				
Rapid onset	51 (33.3)	69 (31.8)	120 (32.4)	0.41
Recent issue	23 (15.0)	34 (15.7)	57 (15.4)	
New neurological deficit (< 24 h)	12 (7.8)	7 (3.2)	19 (5.1)	
Others	67 (43.8)	107 (49.3)	174 (47.0)	
Comorbidities				
Dementia	51 (33.3)	35 (16.1)	86 (23.2)	<0.001
Previous stroke	22 (14.4)	17 (7.8)	39 (10.5)	0.043
Falls in the last 30 days	31 (20.3)	31 (14.3)	62 (16.8)	0.13
Incontinence	48 (31.1)	50 (23.0)	98 (26.5)	0.074
Diabetes	48 (31.4)	66 (30.4)	114 (30.8)	0.84
4AT	(N = 101)	(N = 115)	(N = 216)	
4AT scores, mean (SD)	5.8 (3.51)	4.97 (2.89)	5.36 (3.26)	0.056
4AT ≥4	70 (69.31)	67 (58.26)	137 (58.79)	0.093

MTS, Manchester Triage System. *Unless otherwise noted. ^†^Quantitative variables compared using student’s *t* test; categorical variables using the Chi^2^ test.

Regarding the MTC flow charts and discriminators, patients with a diagnosis of delirium were more likely to be assessed using the ‘behaving strangely’ (15%) flow chart with the ‘rapid onset’ (33.3%) discriminator than the sample as a whole. Regarding comorbidities, people with dementia had nearly three times the odds of having delirium (OR 2.6, 95% CI 1.58, 4.26, *p* < 0.001).

According to the 4AT tool, 84 (38.9%) of the 216 patients had a positive screening result for delirium, while 101 (46.8%) received a medical diagnosis according to DSM-V criteria.

Table 2 shows the accuracy indicators for the 4AT screening test. For the cutoff point proposed in the validation study, the highest sensitivity (94.3%) and specificity (92.0%) were observed in people with dementia. In contrast, these values were lower in older people without dementia and therefore in the screened population as a whole.

**Table 2:** Diagnostic accuracy of the 4AT scale according to two cutoffs, compared to gold standard medical diagnosis using DSM-V criteria in patients with and without dementia.

	Sensitivity (95% CI)	Specificity (95% CI)	PPV (95% CI)	NPV (95% CI)	Youden Index
4AT Cut off ≥ 4					
Total (*n* = 216)	69.3 (78.3, 60.3)	41.7 (50.7, 32.7)	59.3 (68.9, 49.7)	68.4 (76.9, 59.9)	0.11
Dementia (*n* = 64)	94.3 (101.9, 86.6)	92.0 (102.6, 81.4)	58.9 (75.2, 42.6)	25.0 (42.0, 8.0)	0.86
No dementia (*n* = 152)	59.7 (71.9, 47.5)	48.9 (59.2, 38.6)	45.7 (58.1, 33.3)	64.8 (74.7, 54.9)	0.09
4AT Cut off ≥ *3*					
Total (*n* = 216)	85.1 (92.1, 78.2)	66.9 (75.5, 58.3)	52.8 (62.5, 43.2)	71.7 (79.9, 63.5)	0.52
Dementia (*n* = 64)	89.7 (99.3, 80.2)	100	58.3 (73.8, 42.9)	0	0.90
No dementia (*n* = 152)	82.3 (91.8, 72.7)	57.8 (68.0, 47.6)	49.5 (62.0, 37.1)	77.5 (86.13, 68.87)	0.40

CI, confidence interval; PPV, positive predictive value; NPV, negative predictive value.

We analyzed which cutoff point presented the highest diagnostic accuracy for the total sample, observing that a score of 3 points or more on the 4AT scale has the best sensitivity in older people without dementia (82.3%) and therefore in the overall sample (85.1%) (Table 2).

**Figure 2:**
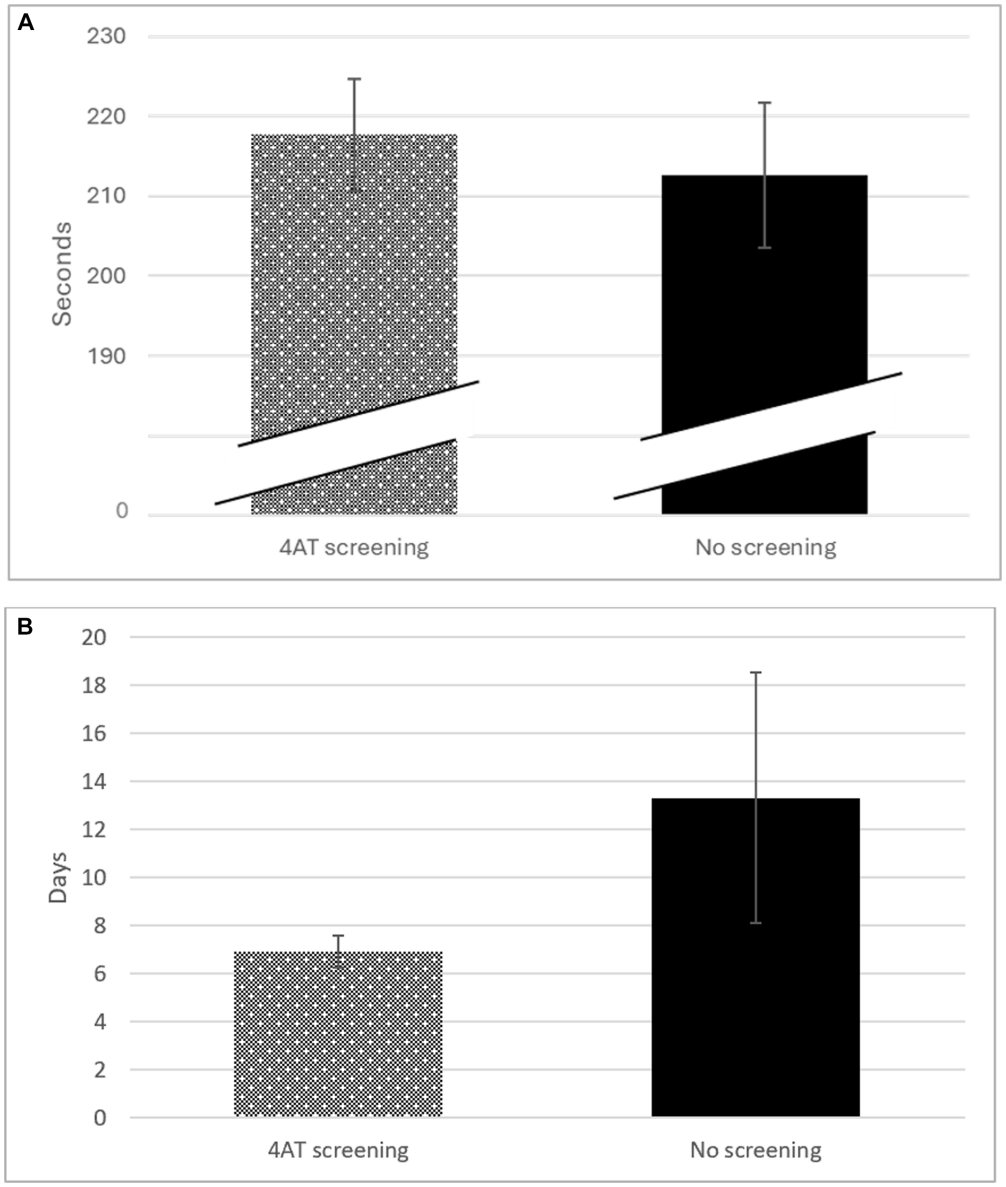
**(A)** Time (Seconds) spent in triage according to performance of screening with the 4AT scale. **(B)** Length of hospital stay according to the performance of screening with the 4AT scale.

Finally, we quantified the time spent performing triage (Figure 2A). The duration of the triage encounters with 4AT screening (mean 218 s SD 104) was similar to triage without screening (mean 213 s SD 113). The scant 5 additional seconds it took to perform screening did not constitute a significant difference (MD 5 95% CI −17, 27; *p* = 0.665).

The average length of hospital stay in participants screened with the 4AT scale episode was half that of patients not screened (mean 6.9 days SD 6.9 vs. 13.3 days SD 44.0, MD 6.4 days; 95% CI −16.9, 4.1; *p* = 0.23), although this difference was not significant (Figure 2B).

## 4 Discussion

Detecting delirium in older people remains a challenge in the ED due to the atypical presentation of many diseases in this population, the existence of several types of delirium, and the failure to implement standardized detection tools in triage settings, despite their availability (22). This research focuses on the detection of delirium in the ED setting in order to improve quality of care in older patients with or at risk of this syndrome. The aim of our study was to determine the accuracy of the 4AT scale performed by nurses in ED triage and to assess the extra time needed for screening in the triage setting. We found that a score of 3 or more points on the 4AT scale accurately identifies people with delirium in ED triage without requiring more time than that spent in conventional triage.

Most studies that analyze delirium in older persons in ED obtain samples of lower age and with a higher percentage of women than those found in our sample. A higher mean age could explain the higher incidence found in the present study. The incidence was 41.4%, which is higher than the 7 to 35% reported elsewhere (7, 18). Within the range of people over 65 years of age or more, older people of 80 years or more present a higher risk of delirium. This aspect is related to the changes inherent to the aging process and to the fact that older people generally have more comorbidity and a greater number of predisposing risk factors. Older age increases almost 3 fold the risk of suffering from delirium with respect to younger older people (23). Indeed, our results showed a higher proportion of males but in pooled analyses the literature does not show a direct association by sex. Finally, the active search through delirium screening for people at higher risk of suffering from delirium could also justify the high prevalence in the population analyzed (24).

The MTS system is a formalized initial assessment system whose main objective is to optimize the waiting time for the first ED assessment by prioritizing acute life-threatening cases (25). Our analysis of MTS is consistent with recent studies showing that priority 3 (urgent) (26) is the priority par excellence assigned to patients with delirium. Regarding the flow chart and discriminators, the most commonly used are ‘unwell adult’ and ‘behaving strangely’ (11). Although this system is general and does not cover all presentation characteristics, recent studies have even shown that it is inadequate as a predictor of severity and mortality (27) especially in older people. More research is needed in this area because nurses with specific knowledge for proper triage (28). Can perform an adequate assessment for the older since the presentation of pathologies may differ with respect to younger populations due to changes in the aging process (12, 29).

The implementation of validated tools specific to older people in the ED setting would improve early detection and minimize the time to optimal treatment (30). The 4AT was specifically designed for routine clinical use in 2011. The Edinburgh Delirium Research Group determined a cutoff of 4 or more points for delirium, and the tool was validated using this cutoff in 2014 (21). However, our results indicate that the 4-point cutoff proposed by the authors presents greater accuracy in people with dementia, while the 3-point cutoff shows greater sensitivity and specificity for the general population of older people (19).

Several meta-analyses have identified greater sensitivity and specificity in the 4AT scale in the older population in the same setting than that obtained in our study (31, 32). However, the age of the samples and the incidence was generally lower than that found in our results, which may be influenced by comorbidity, the presence of cognitive impairment or the cause of delirium. Efforts have been made to determine the most appropriate tool for the detection of delirium in ED, since the CAM scale and the 3D CAM scale are the most widely used and have very good diagnostic accuracy. In ED the need for speed in the assessment both for obtaining the results and for the least time investment should also be an essential aspect to take into account in the assessment procedures, for this reason the 4AT scale is being proposed as the most suitable for the ED setting, (31, 32) even for the detection of cognitive impairment (32). The possible use of different cutoff points increases diagnostic accuracy and therefore detection, so there is a need for studies to analyse different populations and cutoffs, which would help to increase detection through the consideration of comorbidity (20). In addition to designing studies with high methodological quality for greater validity of the results (32).

Finally, some studies suggest that longer hospital stays have short-and long-term effects in patients with delirium (33). The 4AT scale is useful for detecting delirium in the ED, does not require specific training to administer, and requires only about 2 min to perform (19, 32). In our study, the time spent on triage with versus without delirium screening was compared, and no differences were found, with an average of only 5 s more time spent in triage that included screening. These data demonstrate that screening does not interfere in the ED prioritization process (32).

The results obtained on the 4AT scale provide further information to the physician. The teamwork model within the ED makes it difficult to know at what time the physician diagnoses delirium and at what exact moment a treatment is administered by the nursing professional once he/she reads and executes the physician’s order. If the physician has the result of the 4AT scale screening when the physician performs the assessment, he has more information and could perform a faster assessment and therefore prescribe a treatment in less time, which would mean that the nursing professional could administer the treatment earlier. The literature states that early detection enables prompt diagnosis and treatment, and this is associated with shorter and less severe episodes of delirium (34). The mean length of hospital stay following the ED episode was shorter in participants screened with the 4AT scale than in patients who did not undergo screening (6.9 SD 8.9 days vs. 13.3 SD 44.0 days). Evidence suggests that screening reduces the length of hospital stay by 2 days in people diagnosed with delirium (24). Data from our study are highly relevant, because length of stay was half that in patients who were screened versus those who were not. The improvement in hospitalization data could be to considering both the urgency determined by a triage tool and the results of geriatric screening (35). These data are an important advance for clinical care and researchers, particularly where optimized care could prevent the development of delirium and minimize its causes.

### 4.1 Limitations

The impossibility of administering the 4AT scale in patients with reduced awareness, communication barriers, or the absence of a family member as exclusion criteria has reduced the possible size of the final sample. The time from the start of the emergency episode to the start of treatment was not analyzed, as there is no electronic record of this action. Likewise, it was not possible to assess the severity of delirium.

### 4.2 Strengths

In daily clinical practice, delirium screening by nursing staff in older persons at risk of delirium presenting to the ED can assist the physicians in diagnosing delirium without significant increase in time. In addition, it could aid in early detection and treatment, which could prevent further severity, prolonged hospital stays and worse outcomes. The need for longitudinal studies to understand the whole process implies that future studies should address early detection, possible biomarkers and their relationship to severity and consequences.

## 5 Conclusion

The 4AT scale is an accurate screening tool for delirium in older people in the ED. A score of 3 points or more allows people with delirium to be identified in ED triage without consuming more time than that spent in conventional triage. The use of the combined Manchester triage tool, together with the validated 4AT screening, helps to categorize the need for urgent care and shorten hospital admissions.

## Data availability statement

The raw data supporting the conclusions of this article will be made available by the authors, without undue reservation.

## Ethics statement

The studies involving humans were approved by Hospital Francesc de Borja Ethics committee. The studies were conducted in accordance with the local legislation and institutional requirements. Written informed consent for participation in this study was provided by the participants’ legal guardians/next of kin.

## Author contributions

AS-S: Data curation, Formal analysis, Investigation, Writing – original draft. FM-A: Conceptualization, Formal analysis, Writing – review & editing. JS-F: Writing – review & editing. PP-R: Conceptualization, Formal analysis, Funding acquisition, Supervision, Writing – original draft, Writing – review & editing.
